# Performance Evaluation of Multi-Channel Wireless Mesh Networks with Embedded Systems

**DOI:** 10.3390/s120100500

**Published:** 2012-01-05

**Authors:** Jun Huy Lam, Sang-Gon Lee, Whye Kit Tan

**Affiliations:** Department of Ubiquitous IT, Division of Computer & Information Engineering, Dongseo University, Busan, 617-716, Korea; E-Mails: timljh@msn.com (J.H.L.); blueppp@gmail.com (W.K.T.)

**Keywords:** FreeBSD, NanoBSD, multi-channel, wireless mesh network, embedded system, PCEngines alix, 802.11s, static routing, dynamic routing

## Abstract

Many commercial wireless mesh network (WMN) products are available in the marketplace with their own proprietary standards, but interoperability among the different vendors is not possible. Open source communities have their own WMN implementation in accordance with the IEEE 802.11s draft standard, Linux open80211s project and FreeBSD WMN implementation. While some studies have focused on the test bed of WMNs based on the open80211s project, none are based on the FreeBSD. In this paper, we built an embedded system using the FreeBSD WMN implementation that utilizes two channels and evaluated its performance. This implementation allows the legacy system to connect to the WMN independent of the type of platform and distributes the load between the two non-overlapping channels. One channel is used for the backhaul connection and the other one is used to connect to the stations to wireless mesh network. By using the power efficient 802.11 technology, this device can also be used as a gateway for the wireless sensor network (WSN).

## 1. Introduction

Wireless mesh networks (WMNs) have been a popular research topic as wireless devices have become more affordable, which was followed by the mass adoption of wireless technology. Because of its robust, reliable and cost-efficient features, many companies have developed different WMN protocols and products for different application purposes [1–3]. However, no industrial standard existed for WMNs, and thus, these devices were incompatible with each other. Therefore, the Institute of Electrical and Electronics Engineering (IEEE) started the 802.11s Task Group (TG) in July 2004 to unify the research and establish a draft standard for WMNs [4].

The main advantages of the proposed IEEE 802.11s standard are the robustness and the automated generation of the routing table that is used to choose the best path, *i.e.*, whether it is through the proactive or reactive mode. These features of IEEE 802.11s allow the existing nodes to update the new path every time a node is added or deleted from the network, which means node failure will not affect the reliability of the network as much as it did with the previous WMN products.

The widely deployed 802.11 infrastructure can also be used for wireless sensor networks (WSN) to save on the investment cost of deploying a new network and utilize the advantages of 802.11 technologies [5].

The power consumption concern of 802.11 was also eliminated with the power efficient 802.11, where the sensor device is able to run for 5 to 10 years with only 1 AA battery [5]. This device can then be used as a gateway for the WSN. The data rate of the 802.11 technology is also very high compared to the common technologies used in WSN, such as ZigBee, Bluetooth or 802.15.4 [6]. IEEE 802.11s can then be used to form the mesh topology for the WSN.

However, the frequencies used by all of the nodes in the network will interfere with one other if they happen to be in the same or nearby frequency bands. Therefore, minimizing the interference in a WMN or WSN is very important to maximize the performance of the network.

In the Wireless Local Area Network (WLAN) standard, IEEE 802.11 uses the unlicensed frequency bands range from 2.4 GHz to 2.4835 GHz (802.11b and 802.11g) and 5 GHz (802.11a) [7]. To maximize the usage of these limited resources or frequencies, non-overlapping operating channels are used simultaneously. The communication between these channels is less likely to interfere with each other’s operation. For the IEEE 802.11b/g, the operating channels that are separated by at least five channels (or six for Europe) are the non-overlapping operating channels [7]. For example, channels 1, 6 and 11 are the non-overlapping channels. By utilizing 2 or even 3 non-overlapping channels at the same time, simultaneous data transmissions on their respective channels can occurs without causing interference to each other.

The WMN has been applied in commercial products, such as the Open Mesh products, which offer plug-and-play WMN solutions to consumers [1]. However, the proprietary or non standard protocols used in these products make them incompatible with the products from another vendor. According to their official website, even with the existence of incompatibility issues, there are still millions of users around the world [1]. Thus, it is evident that not only is there a vast market for such a product but also research value in building a device that is in accordance with the IEEE 802.11 draft standard.

The open source community then developed a few projects that include a wireless mesh networking ability that adheres to the IEEE 802.11s draft standard in their OS. The Linux community has developed the open80211s project [8], while the FreeBSD has its own implementation [9] that also adheres to the IEEE 802.11s draft standard.

Currently, researchers that are working on test beds or prototypes of WMN based on the Linux open802.11s project, such as the CedtMesh project [10] and the framework implementation by the University of Pisa, Italy [11]. However, the FreeBSD IEEE 802.11s implementation has not been used in any test bed or in any embedded system that acts as a wireless mesh router.

Customized versions of FreeBSD for the embedded system applications are available, such as the pfSense, whose main usage is in firewalls and routers [12]. However, these customized FreeBSD are using an older version of the FreeBSD which does not support the wireless mesh networking. In order to evaluate the FreeBSD IEEE 802.11s implementation, a test bed will be needed.

The main contributions of this paper are the following:

- First, although 802.11s is under standardization, no practical test and performance evaluation has been conducted with WMN reference implementations. We built a test-bed of wireless mesh capable embedded systems that runs on the customized version of FreeBSD OS and conducted extensive performance evaluations. The embedded systems also incorporated two channels that are used to distribute the network load according to its purpose. A series of outdoor performance evaluations had been carried out on the 802.11s protocol used. Comparisons were conducted to demonstrate the advantages of this 802.11s implementation over the conventional wireless distribution system (WDS) interface; moreover, a single channel 802.11s was implemented in a real-time test-bed instead of using simulation.
- Secondly, the path selection mechanism in the FreeBSD IEEE 802.11s implementation is also discussed in this paper based on the two multi-hop WMN tests that were performed on this test-bed. The test results show the flaws in the path selection mechanism used in the protocol (Figures 12 and 13), where the longer path was chosen instead of the shortest path.

The remainder of this paper is organized as follows. Section 2 will explain the wireless mesh network (WMN), hybrid wireless mesh protocol (HWMP), multi-channel WMN and hop count. Section 3 will briefly explain the embedded system implementation. The results and analysis of the performance tests will be discussed in Section 4 and additional discussions will be provided in Section 5. The paper will end with the conclusion in Section 6.

## 2. Background

### 2.1. Wireless Mesh Network (WMN)

Most of the vendors’ proprietary standards were implemented on the Open Systems Interconnection (OSI) layer 3, the network layer [13], with some implementations based on the Linux open802.11s project [8] working on the OSI layer 2 [10,11], the data link layer, because it was built in accordance with the IEEE 802.11s draft standard, while the remaining were implemented on the virtual layer between OSI layers 2 and 3, otherwise known as layer 2.5 [14].

One of the interesting implementations that work on the OSI layer 2.5 is the MIT Roofnet [14]. It is an experimental WMN setup with approximately 50 nodes that placed in apartments in Cambridge, MA, USA. This WMN was used to provide broadband internet access to the users in Cambridge. They used a small form factor computer with Roofnet software to manage the routing information of the WMN, and the best path was then chosen based on this routing information. In Roofnet, the routing protocol SrcRR was used to determine the high throughput paths. The ScrRR Routing protocol was based on Dynamic Source Routing (DSR) [15], and ScrRR uses the expected transmission count metric (ETX) to determine the best possible paths.

The IEEE 802.11s protocol works on OSI layer 2. The Linux open802.11s project and the FreeBSD 802.11s implementation are the only few that follow the draft standard of the IEEE 802.11s. A test bed or prototype of WMN is required to evaluate the network performance of the implementation in a real physical environment. With the help of the test bed, the routing protocol or the routing metric can also be modified to further improve the IEEE 802.11s draft standard, and the resulting modifications can be observed and evaluated.

Song *et al.* [16] implemented their version of an IEEE 802.11-based WMN test bed on July 2007 by altering the existing Wireless Local Area Network (WLAN) functionality. They used Optimized Link State Routing (OLSR) [17] as the routing protocol, and they ran the system on ARM-based FALINUX EX-X5 + EXPCMCIA and x86-based laptop computers with Redhat Linux 9 and Fedora Core 4 distribution as the operating systems (OSs) respectively. Their implementation was tested with multimedia services using Gnomemeeting as the VoIP and video conferencing software and the workability was confirmed to be successful [16].

Granelli *et al.* [18] fabricated another implementation of IEEE 802.11-based WMN test bed and published their research in 2010. In terms of hardware, PCEnglines ALIX 2C2, PCEngines WRAP IE and the Gateworks Cambria GW2358-4 were used in their implementation. Their system ran on an OpenWRT platform and was based on the Roofnet platform that works on OSI layer 2.5 which was explained earlier. In their implementation, they were able to perform simultaneous transmissions, and the intra-path interference was reduced due to the use of the non-overlapping channels. They developed the Interference and Traffic Aware Channel Assignment (ITACA) algorithm that considers the traffic condition and interference patterns for better channel assignment because their implementation used multiple radios per mesh point (MP).

In 2009, Garroppo *et al.* [11] implemented a system that is based on the IEEE 802.11s draft standard. Their prototype was able to run in both of HWMP routing protocol's modes of operations, which are the proactive and reactive modes and the path selection based on the routing metric was evaluated. Laptop and desktop computers were used in their implementation along with Linux as the OS and C as the programming language of the software framework, and the Multiband Atheros Driver for Wi-Fi (MadWifi) [19] was used to create the wireless interfaces in the station, access points (APs) or WDSs.

The test bed developed by Ghannay *et al.* [20] also implemented a multi-radio, multi-channel WMN, which was used to evaluate and compare the routing metrics, such as the Weighted Cumulative Expected Transmission Time (WCETT), Metric of Interference and Channel-switching (MIC), Interference Aware routing metric (iAWARE). The throughput analysis can be used to indicate the effectiveness of the applied routing protocol or routing metric. In their research, the pros and cons of each evaluated routing metric were identified by comparing the throughputs.

We built an IEEE 802.11s-based WMN test bed with the embedded systems, PCEngines ALIX 3d2. We used the HWMP routing protocol in our implementation in conjunction with the FreeBSD IEEE 802.11s implementation.

### 2.2. Hybrid Wireless Mesh Protocol (HWMP)

In addition to the frequency bands used, the routing protocol is another important factor that dramatically affects the network performance. The Hybrid Wireless Mesh Protocol (HWMP) is used as the mandatory routing protocol to adhere to the IEEE 802.11s draft standard. In this protocol, the network is allowed to operate in both proactive and reactive modes.

In the proactive mode, the MPs communicate with each other with the help of the root and the tree topology will be built with either the Route Request (RREQ) or the Root Announcement (RANN) mechanism. The RREQ message will be broadcasted by the root MP or the root mesh portal to create the paths between the root MP and all the other MPs in the WMN, while the RANN message distributes the routing information to reach the root MP [4]. The tree will then be created and maintained for all the MPs in the WMN to reach the root MP because it contains the information for the tree topology [21].

The reactive mode allows the MPs to communicate with each other through the path created on-demand by the network and no root MP is required [4]. If there is no root MP available in the WMN, the WMN will be able to operate solely on the reactive mode. The RREQ message will be broadcasted by the MP as the route discovery process begins if it needs a path to its destination MP and the destination MP is not in its routing table [21]. The received RREQ message will be used to create a path to the source or update the path if the RREQ consists of the same or greater sequence number with an improved metric [4]. The destination MP or any intermediate node that is connected to the destination MP will respond to the RREQ with a unicast RREP message [21]. However, the initial latency for the path formation that lasts until the first data packet is received by the MP that initiated the route discovery process [21].

A root portal is required for the system to operate in the hybrid mode [21]. Because the root portal has the information for all MPs in the WMN when the registration mode is used, it can accelerate the route discovery process by forwarding the data frames to the MP that wants to form a path with the other MP that is in the same WMN [21]. This information will be sent to the requesting MP along with the signal that these two MPs are in the same WMN, and thus, a route discovery will be initiated and the optimal path between these two MPs will be formed [21]. This hybrid mode will improve the conventional reactive mode with the information obtained from the proactive mode [21]. The initial latency in the path settings is then eliminated [21].

To find the best path for route formation, the link cost for each link in the WMN had to be computed. The link with the lowest metric value will be the chosen path for the transmission of the data [4]. This metric value can be found in the metric field of the RREQ, Route Reply (RREP) and RANN messages, and will be propagated to build or update the path [4]. In IEEE 802.11s, the airtime link metric was defined as the default link metric computation method for the path selection [4]. This airtime link metric is a radio-aware routing metric. By transmitting a frame over the particular wireless link, the metric can measure the amount of channel resources consumed [4].

### 2.3. Multi-Channel Wireless Mesh Network (M-WMN)

In a conventional wireless networking device, the operation is only enabled on 1 channel. There are two methods that can be used to implement the multi-channel wireless mesh network; multiple radios on the physical layer (PHY) or using the channel switching capability of the device [4]. In this embedded system implementation, the former method in which two wireless network devices with a single radio PHY each were used. However, the two channels of the wireless network devices in this embedded system solution will be used for different purposes.

### 2.4. Hop Count

**Figure 1.**
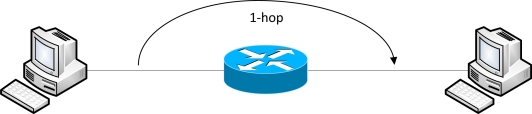
1-hop connection.

According to [22,23], the hop count refers to the number of intermediate routers the data will pass through, which clears the misconception that a direct connection between a point-point link in a transmission path is a 1-hop connection. A direct connection does not go through an intermediate router, and thus, it is not a 1-hop connection. However, when an intermediate router exists between them, this can be regarded as 1-hop connection. Figure 1 shows a simple 1-hop connection.

## 3. The Embedded System Implementation

The embedded systems used in these tests are the PCEngine ALIX 3d2 with a 4 GB Sandisk Ultra Compact Flash (CF) card as the storage medium for the OS and the software. Two Compex WLM54G23 wireless network cards that support the 802.11a/b/g on the physical layer and based on the Atheros AR 5414 chipset were added to the miniPCI slots in each of the embedded systems along with two Sub-Miniature version A (SMA) omni-directional antennas. The specifications of the PCEngline ALIX 3d2 are as follows [24]:

**Table:** 

• CPU:	500 MHz AMD Geode LX800
• DRAM:	256 MB DDR DRAM
• Storage:	CompactFlash socket
• Power:	DC jack or passive POE, min. 7 V to max. 20 V
• Three LED indicators	
• Expansion:	2 miniPCI slots, LPC bus
• Connectivity:	1 Ethernet (Via VT6105M 10/100)
• Input/Output:	DB9 serial port, dual USB
• Board size:	100 × 160 mm
• Firmware:	tinyBIOS

**Figure 2.**
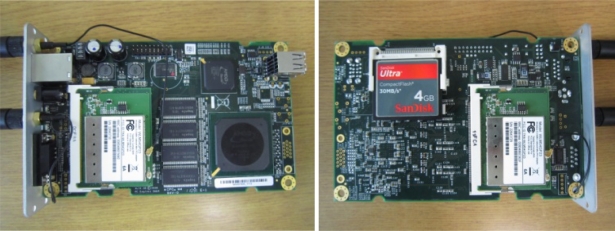
The (left photo) top view and (right photo) bottom view of the embedded system.

A picture of the embedded system with two wireless network cards and an installed CF card is shown in Figure 2 below.

NanoBSD was used to build the embedded version of the FreeBSD for this test bed implementation. Two configurations need to be created to build the FreeBSD image with NanoBSD, namely, the configuration of the FreeBSD kernel and NanoBSD. After the image building process, the image will be copied to the CF card. The embedded version of FreeBSD then runs on the embedded system in read-only mode.

Some system files need to be modified after the OS has been copied to the CF card such that the embedded system boots with the wireless mesh interface and AP interface instead of the default station interface. Further configurations were added to the system files, such as the Internet Protocol (IP) address settings for each of the embedded systems and installation of the performance evaluation software in the embedded system; furthermore, the Dynamic Host Configuration Protocol (DHCP) was also configured so that the stations will be able to connect to the WMN with ease.

In the system implementation, two wireless network cards were installed in each of the systems to distribute the network resources for different purposes. Two non-overlapping channels were used in the test: channel 11 for the backhaul connection and channel 6 for the stations to connect to the WMN. In this case, the wireless network cards will be initialized to work in the different mode and channel. The wireless mesh interface will be assigned to channel 11, and the AP interface will be assigned to channel 6. These two wireless network cards were then bridged together to communicate with each other.

The WMN implementation of the FreeBSD can configure the topology automatically (default) or manually. To configure the topology of the WMN manually, the wireless local area network access control list (wlan_acl) had to be enabled during the NanoBSD image building phase or manually enabled with the help of a command [25].

Further explanations on how to configure the interfaces to work as the wireless mesh interfaces, the routing implementation of the IEEE 802.11s under FreeBSD and how to implement a routing protocol can be found in the paper by Paolo [9].

## 4. Experimental Results and Discussion

### 4.1. Experimental Results

Iperf [26] was used as the performance evaluation tool to obtain the bandwidth result of the backhaul WMN and the stations that make use of the WMN. To evaluate the performance of the embedded system implementation, a series of tests were performed and the results were compared as described below. IEEE 802.11g is used as the wireless technology of the physical layer (PHY) in all the tests done is. Because the IEEE 802.11s is the routing protocol that works on OSI layer 2, IEEE 802.11g is needed to handle the tasks on PHY layer or the OSI layer 1. The setup parameters are listed as follows:

- Default transmission power:
  ○ The wireless mesh interface: 24.5 mW
  ○ The AP interface: 24 mW
- Channels used: Channels 6 and 11 of 802.11g

**Figure 3.**
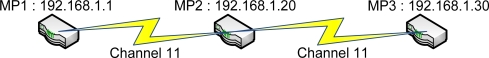
The 1-hop backhaul WMN connection (test 1).

**Figure 4.**
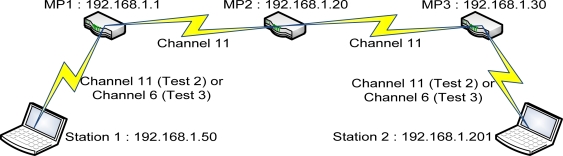
The 2 mobile stations that are connected to each other through the backhaul WMN.

The first three tests use static routing to test the multi-hop network performance because the distance is not long enough to do a practical test. The details of the three tests are given in the following:

- Test 1: test the 1-hop backhaul WMN connection as shown in Figure 3.
- Test 2: two mobile stations running non-FreeBSD OS were connected to the same backhaul WMN at each end through the same channel, as shown in Figure 4.
- Test 3: similar to test 2 but it will be using two non-overlapping channels instead of the same channel for the backhaul and the station connection as shown in Figure 4.

All of these tests were performed in an outdoor environment with interference coming from APs and stations within the test range. On average, approximately 30 APs were detected within the communication range of the embedded systems by utilizing the Linux scanning command *iwlist wlan0 scan* on a laptop computer that is running Ubuntu 11.10. Given these conditions, a significant amount of interference exists and renders the result unstable.

**Figure 5.**
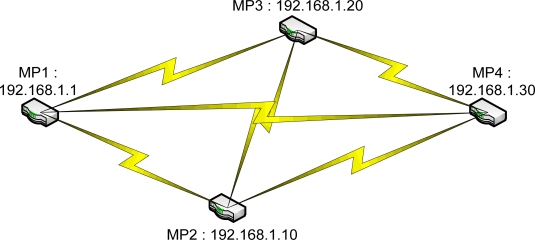
The mesh topology used to determine the path selected in the dynamic routing mode.

**Figure 6.**
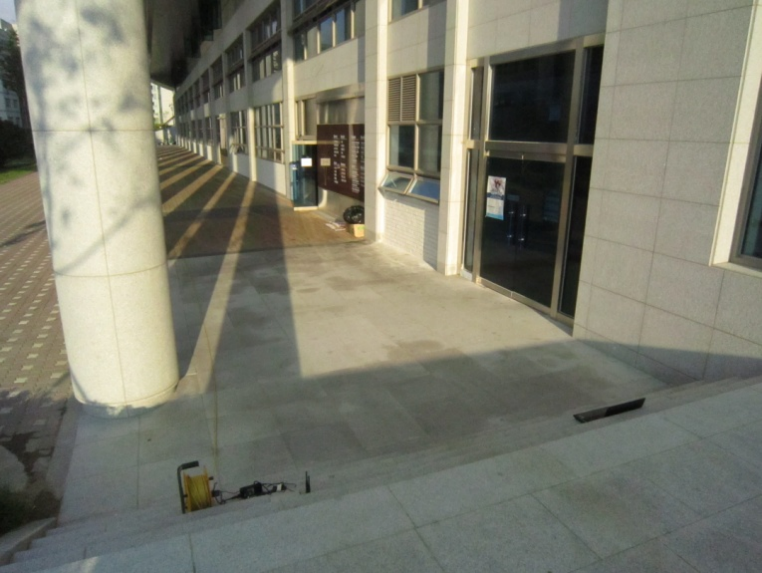
Photo of the actual field test where (**left**) MP1 and (**right**) station 1/laptop are located.

**Figure 7.**
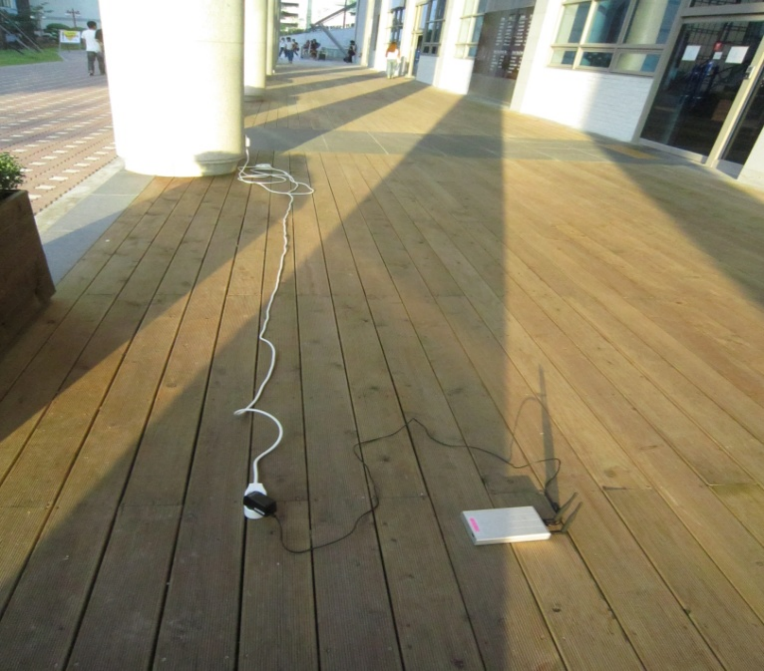
Photo of the actual field test where MP2 is located in between MP1 and MP3.

**Figure 8.**
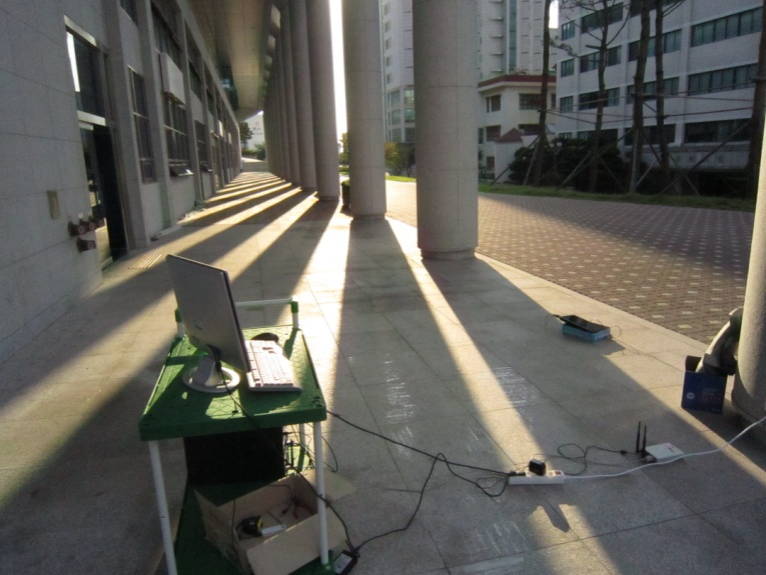
Photo of the actual field test where (**left**) station 2/desktop and (**right**) MP3 are located.

The last two tests were performed to show the path selected by the FreeBSD IEEE 802.11s implementation in the default mode or the dynamic routing mode instead of the static routing used in the previous tests. A simple mesh topology was used to evaluate the path selection mechanism of the 802.11s implementation as shown in Figure 5. The photos of the field test indicate the location of each MP and station (Figures 6–8).

**Figure 9.**
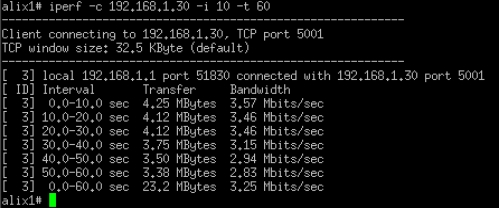
Sample bandwidth results of the MP1-MP3 connection.

**Figure 10.**
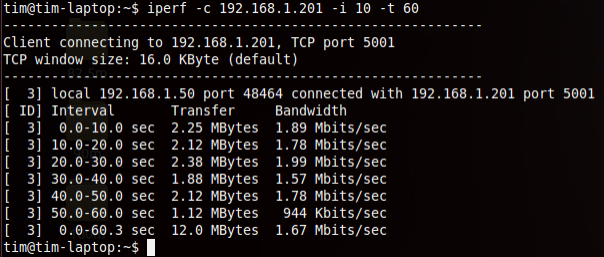
Sample bandwidth results for the stations that were connected to the WMN through the same channel with the backhaul connection.

**Figure 11.**
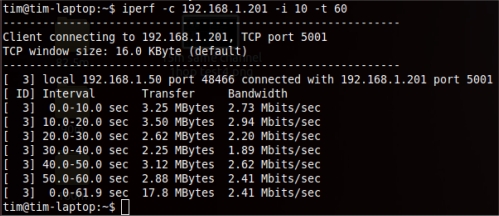
Sample bandwidth results for the stations that were connected to the WMN through a different channel with the backhaul connection.

In the first three tests, each range was evaluated with 8 trials, and an average value was calculated to ensure the consistency of the results because the tests were performed outdoors where a significant amount of interference exists. Each trial was run for 60 seconds with a 10-second interval as the input to the iperf. The sample bandwidth results are shown in Figures 9–11. The results were then tabulated and compared.

The range value in the tables indicates the distance between MP1 and MP3 where MP2 will be located in the middle of the 2 MPs. For example, for the 15 m test, the MP2 will be located 7.5 m away from both MP1 and MP3. This placement will be applied to tests 2 and 3 as well. The results of tests 1, 2 and 3 are tabulated in Tables 1–3, respectively.

From Table 4, the comparison between the bandwidth test of the backhaul connection and the stations that are connected to the backhaul connection at each end through two non-overlapping channels, the bandwidth was only degraded by approximately 12.5% to 24.5%. In this scenario, the communication between the two stations at each end involves two additional hops, which indicates that the performance degradation was much lower compared to that of the WDS, which degrades by approximately 50% with each additional hop. An exact comparison is not possible because the FreeBSD WMN implementation does not allow legacy stations to connect to the WMN without the help of the second channel.

To determine how the usage of two non-overlapping channels helps the network performance, a second comparison was performed with the same channel for both the wireless mesh interface and the AP interface in test 2 and different channels in test 3. The result is shown in Table 5. The network performance of test 2, which uses the same channel, shows instability: there is an improvement of up to 164% in the data rate when the usage of the same channels was changed to the two non-overlapping channels. With the help of the non overlapping channel, the load balancing ability of the two channels will be maximized.

In the last test, which used dynamic routing to analyze the path chosen by the routing protocol implemented in the FreeBSD 802.11s implementation, the embedded systems were placed in a simple mesh topology in which the distance between MP1 and MP4 is approximately 60 m, and the distance between MP2 and MP3 is approximately 10 m. Two trials were performed to analyze whether the path changes over time.

The routing information was obtained from each node after they were run with the established paths. This information was then visualized in diagrams, and the path chosen at each node is represented by different line types—the straight line, dotted line or the dashed line—as stated below. The value beside the line represents the metric value obtained from the test.

**Figure 12.**
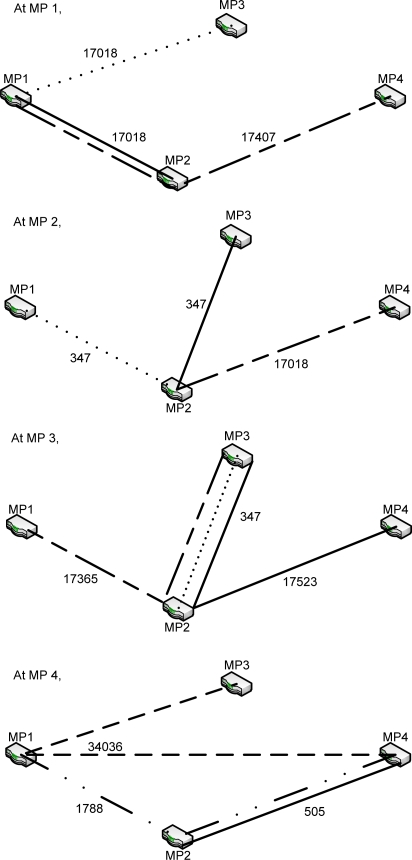
The path selected at each node (Trial 1).

Based the result of the first trial in the last test, as shown in Figure 12, some flaws in the metric calculation of the FreeBSD 802.11s implementation can be observed. The metric calculation does not account for the hop count because the more expensive path was chosen at MP3 and MP4. At MP3, the routing protocol actually chose to hop through MP2 to reach MP1 or MP4, even though the direct path to MP1 or MP4 might offer better network performance. The same was true for MP4, where MP4 chose to hop through MP1 to reach MP3. This phenomenon indicates that the metric calculation can be improved by considering the hop count of the path.

**Figure 13.**
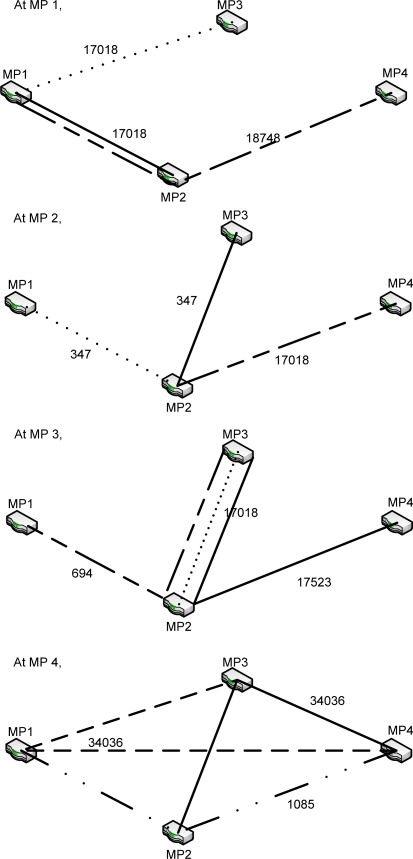
The path selected at each node (Trial 2).

In the second trial of the last test, as shown in Figure 13, the inefficient paths were chosen again, as observed from the information obtained at MP3 and MP4. Similar results were observed for the MP1, MP2 and MP3 but worse paths were selected at MP compared to the previous trial. MP4 communicates with MP2 through MP3, and MP3 communicates through MP1. These two paths are obviously not the best paths because they require travelling a longer distance and an additional hop, compared to the direct path.

### 4.2. Discussion

The second channel of the embedded system that is used to connect the stations to the WMN backhaul connections can be further modified to suit the needs of various unique situations. For example, sensors can be used to connect to the WMN backhaul connection using ZigBee, Bluetooth or Wi-Fi, depending on the application’s needs. These connections make it possible to implement the network that incorporates the advantages of two or more wireless technologies: the hybrid version of WMN and WSN. The advantage of Wi-Fi is the ability to transmit at a high data rate and the long communication range is suitable for the backhaul connection while the advantage of ZigBee, Bluetooth or the power efficient 802.11 is much lower consumption which can be used for the WSN [5,6].

## 5. Conclusions

There are two main advantages of implementing a multi-channel WMN. The compatibility issue was overcome in this implementation by building one of the wireless interfaces as a normal AP. This implementation allows the legacy stations to connect to the WMN independent of the OS used. The second advantage of the implementation is the load balancing ability. By using two non-overlapping channels for the two different purposes, it is possible to distribute the load for the backhaul system and the connection between the stations and the AP.

The performance improves significantly when using two non-overlapping channels for the different purposes when compared to the theoretical degradation of WDS. Moreover, the usage of two non-overlapping channels also shows significant improvement in network performance compared to the test where the same channel was used for both the wireless mesh interface and the AP interface.

The dynamic routing mode of the FreeBSD 802.11s implementation also highlights some flaws in the path selection method because the more expensive path was chosen instead of the more direct path which, in theory, should offer the best metric.

Therefore, this result indicates that the FreeBSD 802.11s implementation can be further improved with a better routing metric. The improved channel reservation and security features such as the encryption and authentication can also be added to this implementation because the current FreeBSD 802.11s implementation does not include such features [9]. By implementing the embedded system with the power efficient 802.11, the system can also be used as a gateway for WSN.

## Figures and Tables

**Table 1.** The bandwidth results for the MP1–MP3 connection.

**Range**	**Throughput (Mbps)**								

	**Trial 1**	**Trial 2**	**Trial 3**	**Trial 4**	**Trial 5**	**Trial 6**	**Trial 7**	**Trial 8**	**Average**

**15 m**	3.16	3.01	3.36	3.24	2.71	3.25	3.22	2.98	3.116
**30 m**	2.33	2.85	2.99	2.68	2.46	2.38	2.41	1.70	2.475
**45 m**	2.28	2.78	2.82	2.42	2.40	2.57	2.53	2.65	2.556
**60 m**	1.41	1.69	1.93	1.73	1.80	1.57	1.73	1.41	1.659
**75 m**	1.89	2.15	1.87	1.55	1.82	2.08	2.12	2.34	1.978
**82.5 m**	2.01	2.41	2.14	2.01	1.98	2.18	2.14	2.24	2.139
**90 m**	1.84	1.87	1.50	1.93	1.53	2.07	2.18	2.04	1.870
**100 m**	2.23	2.35	2.38	2.20	2.20	2.22	2.26	2.17	2.251

**Table 2.** The bandwidth results for the station 1–station 2 connection that uses the same channel for both the mesh and AP interfaces.

**Range**	**Throughput (Mbps)**								

	**Trial 1**	**Trial 2**	**Trial 3**	**Trial 4**	**Trial 5**	**Trial 6**	**Trial 7**	**Trial 8**	**Average**

**15 m**	1.60	1.93	1.67	1.65	2.41	2.16	2.18	2.23	1.979
**30 m**	1.71	1.75	1.42	1.75	1.37	1.28	1.17	1.20	1.456
**45 m**	1.13	0.71	0.48	0.62	0.57	0.68	0.64	1.08	0.738
**60 m**	1.3	0.93	1.33	1.29	1.28	1.14	1.54	1.39	1.274
**75 m**	0.43	0.40	0.63	1.37	1.39	1.39	1.31	1.66	1.072
**82.5 m**	1.26	1.00	1.21	1.39	1.17	1.11	1.01	0.98	1.141
**90 m**	1.44	1.57	1.55	1.53	1.19	1.07	1.35	1.23	1.366
**100 m**	1.28	1.63	1.66	1.30	1.37	1.42	1.39	1.63	1.460

**Table 3.** The bandwidth results for the station 1–station 2 connection that uses different channels for the mesh and AP interfaces.

**Range**	**Throughput (Mbps)**								

	**Trial 1**	**Trial 2**	**Trial 3**	**Trial 4**	**Trial 5**	**Trial 6**	**Trial 7**	**Trial 8**	**Average**

**15 m**	2.21	2.39	2.39	2.35	2.41	2.30	2.24	2.55	2.355
**30 m**	1.91	2.13	1.92	1.87	1.79	1.77	1.87	1.83	1.886
**45 m**	1.98	1.78	1.97	2.06	2.07	1.84	1.82	2.04	1.945
**60 m**	1.30	1.52	1.32	1.49	1.57	1.45	1.56	1.41	1.453
**75 m**	1.48	1.81	1.50	1.58	1.61	1.65	1.62	1.35	1.575
**82.5 m**	1.67	1.62	1.62	1.64	1.70	1.71	1.71	1.60	1.659
**90 m**	1.33	1.23	1.53	1.46	1.43	1.57	1.37	1.34	1.408
**100 m**	1.69	1.69	1.62	1.73	1.62	1.72	1.69	1.70	1.683

**Table 4.** A comparison between test 1 and test 3.

**Range**	**Average Throughput (Mbps)**		

	**MP1–MP3**	**Station 1–station 2**	**Degradation rate**

**15 m**	3.116	2.355	24.43%
**30 m**	2.475	1.886	23.79%
**45 m**	2.556	1.945	23.91%
**60 m**	1.659	1.453	12.44%
**75 m**	1.978	1.575	20.25%
**82.5 m**	2.139	1.659	22.44%
**90 m**	1.870	1.408	24.72%
**100 m**	2.251	1.683	25.26%

**Table 5.** A comparison between test 2 and test 3.

**Range**	**Average Throughput (Mbps)**		

	**Same channel**	**Different channel**	**Improved rate**

**15 m**	1.979	2.355	19.02%
**30 m**	1.456	1.886	29.53%
**45 m**	0.738	1.945	163.51%
**60 m**	1.274	1.453	13.98%
**75 m**	1.072	1.575	46.94%
**82.5 m**	1.141	1.659	45.43%
**90 m**	1.366	1.408	3.02%
**100 m**	1.460	1.683	15.89%
